# Roxadustat, a HIF-PHD inhibitor with exploitable potential on diabetes-related complications

**DOI:** 10.3389/fphar.2023.1088288

**Published:** 2023-02-10

**Authors:** Tingting Fang, Congcong Ma, Zhanming Zhang, Luning Sun, Ningning Zheng

**Affiliations:** ^1^ Department of Pathophysiology, College of Basic Medical Science, China Medical University, Shenyang, Liaoning, China; ^2^ Pharmaceutical Sciences, China Medical University-The Queen’s University of Belfast Joint College, Shenyang, Liaoning, China

**Keywords:** roxadustat, diabetes mellitus, diabetic nephropathy, hypoxia-inducible factor, diabetic cardiomyopathy (DCM)

## Abstract

Diabetes mellitus (DM) is a group of metabolic diseases caused by absolute or relative deficiency of insulin secretion and characterized by chronic hyperglycemia. Its complications affect almost every tissue of the body, usually leading to blindness, renal failure, amputation, etc. and in the final stage, it mostly develops into cardiac failure, which is the main reason why diabetes mellitus manifests itself as a high clinical lethality. The pathogenesis of diabetes mellitus and its complications involves various pathological processes including excessive production of mitochondrial reactive oxygen species (ROS) and metabolic imbalance. Hypoxia-inducible Factor (HIF) signaling pathway plays an important role in both of the above processes. Roxadustat is an activator of Hypoxia-inducible Factor-1α, which increases the transcriptional activity of Hypoxia-inducible Factor-1α by inhibiting hypoxia-inducible factor prolyl hydroxylase (HIF-PHD). Roxadustat showed regulatory effects on maintaining metabolic stability in the hypoxic state of the body by activating many downstream signaling pathways such as vascular endothelial growth factor (VEGF), glucose transporter protein-1 (GLUT1), lactate dehydrogenase (LDHA), etc. This review summarizes the current research findings of roxadustat on the diseases of cardiomyopathy, nephropathy, retinal damage and impaired wound healing, which also occur at different stages of diabetes and greatly contribute to the damage caused by diabetes to the organism. We attempts to uncover a more comprehensive picture of the therapeutic effects of roxadustat, and inform its expanding research about diabetic complications treatment.

## Introduction

**FIGURE 1:**
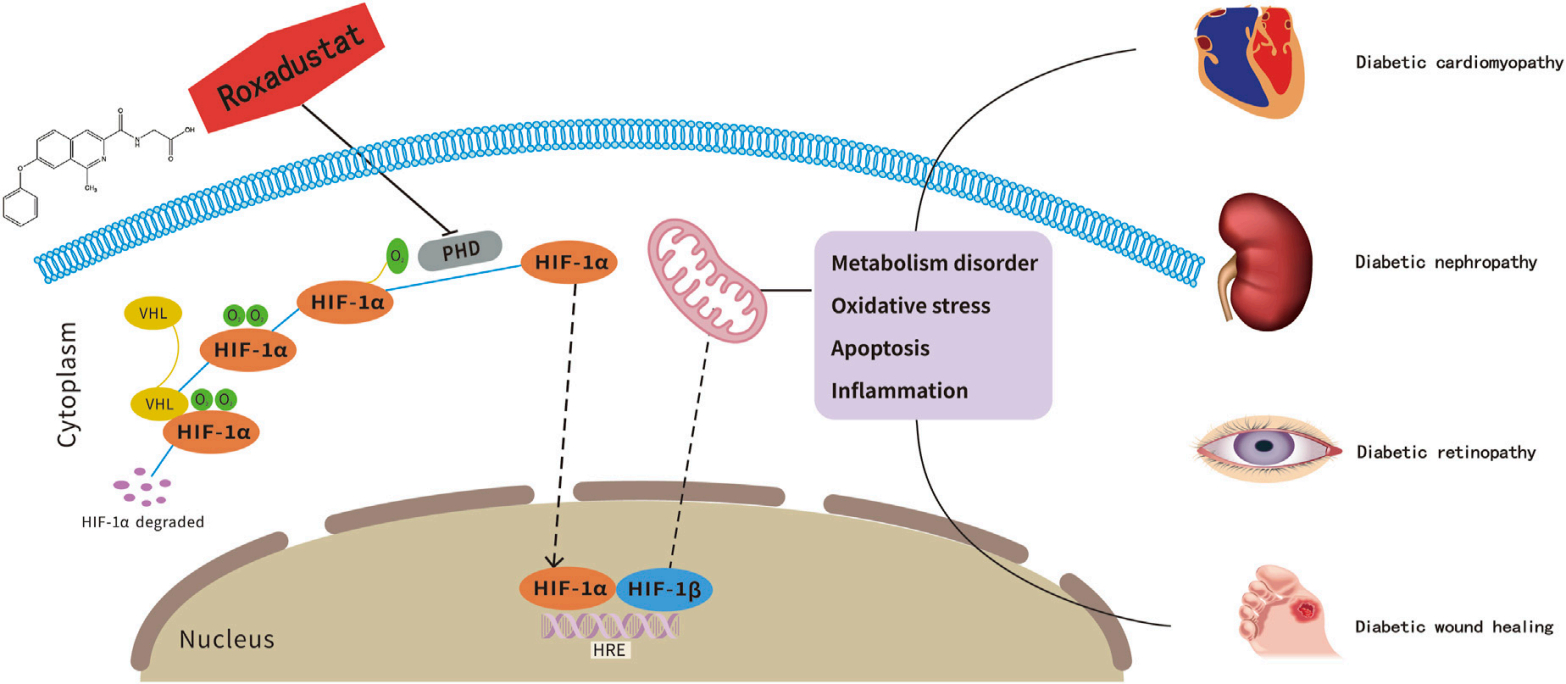
Roxadustat, a potential therapeutic drug for diabetes-related complications treatment. The pathogenesis of diabetes-related complications is related to the HIF-1α-mediated metabolic imbalance. As an inhibitor of HIF-PHD, roxadustst shows potentially therapeutic effects on diabetic complications.

Diabetes mellitus (DM) is a metabolic disease mainly characterized by sustained hyperglycemia (Wojciechowska et al., 2016). It is manifested by insulin resistance or failure to produce enough insulin to regulate blood glucose. According to the latest estimates of the International Diabetes Federation Diabetes Atlas, the number of people with DM will exceed 450 million worldwide, which is also expected to reach 693 million by 2045 (Cho et al., 2018). DM and diabetes-related complications are amongst the leading causes of mortality worldwide (Patel et al., 2022). DM has more than 100 kinds of complications, which has the most known complications of diseases. Clinical data show that about 10 years after the onset of DM, 30%–40% of diabetic patients will develop at least one complication, and once the complication occurs, it is difficult to reverse it with drug treatment, which greatly increases the patient’s morbidity and mortality. Improper blood glucose control will aggravate the manifestation of DM complications, and the interaction between the numerous complications of DM will further destabilize blood glucose, thus forming a vicious circle between DM complications and the control of glucose (Atif et al., 2018).The central role of hypoxia in the development of DM and its complications has been widely demonstrated. Hypoxic state and inhibition of HIF-1α expression are major contributors to the development of impaired glucose uptake and insulin resistance in cardiomyocytes of DM patients (Catrina and Zheng, 2021). However, humans living at a higher altitude are less prone to suffer from impaired glucose homeostasis as well as type 2 diabetes mellitus (T2DM), which might at least partly be explained by lower oxygen availability at higher altitudes (Koufakis et al., 2019). In adipose tissue, a potential mechanism of hypoxia-induced decrease in blood glucose levels is related to the HIF-1α system mediating the switch from aerobic to anaerobic metabolism (Trayhurn, 2013). Overexpression of mitochondrial ROS is another central pathogenic mechanism of diabetic-related complications (Ridzuan et al., 2016). In a sustained hyperglycemic state, increased expression of HIF-1 attenuates the overproduction of mitochondrial ROS and plays a protective role in the model of mouse kidney epithelial cells against apoptosis (Zheng X. et al., 2022). The above results suggest that hypoxia not only causes metabolic disorders but also maintains stable glucose levels to some extent. And the factors of how hypoxia affects metabolic homeostasis may be related to the duration and severity of hypoxia, the activity of HIF-1α, and the downstream pathways activated by it under different conditions. Hypoxia and the HIF signaling pathway are the pivotal influencing factors in diabetes-related complications (Catrina and Zheng, 2021). HIF-1 is a heterologous dimeric transcription factor consisting of two subunits: HIF-1α and HIF-1β(Ke and Costa, 2006), which are widely expressed in mammalian cells. Under normoxic conditions, HIF-PHD binds to HIF-1α and promotes its degradation. Under hypoxic conditions, reduced hydroxylase activity promotes stabilization and translocation of HIF-1α protein to the nucleus, thus allowing for a transcriptional response to low oxygen levels (Fandrey, 2004; Akizawa et al., 2020a). Roxadustat is a novel oral anti-renal anemia drug developed by FibroGen, which was approved for marketing in China in December 2018 (Dhillon, 2019). As an inhibitor of HIF-PHD, roxadustat can reversibly bind to it and significantly reduce its activity, and then inhibit HIF-1α degradation and improve transcriptional activity of HIF-1α and downstream pathway (Besarab et al., 2015; Jain et al., 2016). Except stimulating the synthesis of endogenous erythropoietin for anti-renal anemia, roxadustat can also regulate iron metabolism (Haase, 2010). Activated HIF-1α can promote glycolysis by regulating its downstream target proteins such as VEGF (promotes angiogenesis), GLUT1 (promotes glucose transport), LDHA (promotes glycolysis), etc. Whether roxadustat can play some positive therapeutic role in DM by regulating HIF-1α is an important aspect to develop and expand its clinical application. Here, we summarized the progress of research on roxadustat regulating metabolic stability in diseases of heart, kidney, retinopathy and wound healing, and sorted out the existing studies in diabetes to provide clues for advanced exploration of roxadustat in diabetic related complications (Figure 1).

### Roxadustat and diabetic cardiomyopathy

Diabetic cardiomyopathy (DCM) is a descriptive terminology used to define myocardial dysfunctions in the presence of DM and the absence of coronary artery disease, valvular heart disease, and other conventional risks for cardiovascular diseases (e.g., hypertension, dyslipidemia, and alcoholism (Bai et al., 2016). As the prevalence of DM continues to rise, the DCM increases in parallel.The pathophysiology of DCM is complex and multifactorial. It is widely accepted that DCM is associated with abnormal energy metabolism、oxidative stress、apoptosis、and inflammation, which ultimately lead to left ventricular diastolic and systolic dysfunction (Huynh et al., 2014; Evangelista et al., 2019; Li et al., 2020a; Ritchie and Abel, 2020). As we know, the normal adult heart consumes a large amount of energy and uses a variety of substrates to produce ATP. Seventy percent of total energy is derived from mitochondrial oxidative phosphorylation of fatty acids, with most of the rest derived from glucose oxidation (Bayeva et al., 2013). In diabetic myocardial tissue, glucose transporter protein-4 (GLUT4) activity is reduced, which decreases transmembrane transport of glucose into the cardiomyocyte, and cardiomyocyte glucose uptake is reduced subsequently affecting energy metabolism (Huynh et al., 2014). At the same time, fat β-oxidation increases, and triglycerides and free fatty acids accumulate in cardiomyocytes, resulting in increased oxygen consumption and ROS production in myocardial tissue, which causes cardiomyopathy by a variety of downstream actions. (Carley and Severson, 2005; Mansor et al., 2016). The increased mitochondrial fatty acid uptake and β-oxidation in diabetic hearts may exceed the capacity of mitochondrial respiration and induce an accumulation of toxic lipid metabolites, resulting in cardiac lipotoxicity and mitochondrial dysfunction which will stimulate ROS generation and cardiac oxidative stress and inflammation (Jia et al., 2016). In the end-stage of DCM, it often leads to left ventricular diastolic and systolic dysfunction, and finally ends in heart failure. Whereas the main cause of dysfunction in heart failure is mismatching between persistent hypoxia and diminished HIF-1 signaling (Oka et al., 2014). A key element of HIF activation is the promotion of glycolysis and suppression of fatty acid oxidation, reprogramming cardiac substrate metabolism (Mansor et al., 2016). High dependence on fatty acids in diabetic patients has been found to inhibit glycolysis, leading to a downregulation of myocardial succinate in hypoxia, eventually reducing the stability of HIF-1α(Dodd et al., 2018). Intracellular hyperglycemia also causes excessive ROS production by increasing electron leak from the mitochondrial electron transport chain to further induce apoptosis. Given that most of the ATP required by the heart is produced by mitochondrial oxidative phosphorylation, oxygen plays a crucial role in cardiac energy metabolism (Knutson et al., 2021). The increased ROS induces further mitochondrial dysfunction and decreases fatty acid oxidation capacity, resulting in lipid accumulation, fibrosis, diastolic dysfunction and heart failure in individuals with diabetes (Jia et al., 2018). HIF-1α has been shown to stimulate the rapid growth of myocardial collateral vessels by activating VEGF, which contributes to cell survival after myocardial infarction (Banai et al., 1994; Lee et al., 2000). Four weeks after coronary ligation leads to proper activation of HIF-1α signaling is essential for myocyte survival after myocardial ischemia and heart failure (Sousa Fialho et al., 2019). Mouse and rat models of T1DM and T2DM display systemic inflammation relatively early in disease progression (Nagareddy et al., 2013). DCM is associated with augmented inflammation in the heart (Guo et al., 2022). Studies have shown that the increased expression of HIF helps to alleviate the inflammatory response of cardiomyocytes (Yu et al., 2020). Roxadustat has been shown to achieve myocardial protection through the activation of HIF-1α.It was shown that in β-hydroxybutyric acid (β-oHb)-treated cardiomyocytes, roxadustat stabilized the expression of HIF-1α, and enhanced the expression of GLUT1 and GLUT4, thereby enhancing glycolysis in cardiomyocytes and partially ameliorating cardiomyocyte death caused by elevated β-oHb levels (Ma et al., 2021). In roxadustat pretreated ischemic cardiomyocytes, roxadustat enhanced anaerobic glycolysis and maintained ATP production under anaerobic conditions, thus achieving a significant reduction in myocardial ischemia-reperfusion injury in mice (Deguchi et al., 2020). Roxadustat improved the energy supply and glycerophospholipid metabolism in cardiomyocytes, possibly through upregulating the HIF-1α-targeted genes of LDHA and PDK1 in glycolysis and CHK-α, respectively (Long et al., 2023). Roxadustat could protect cardiomyocytes against DOX-induced apoptosis and ROS production in line with the upregulation of HIF-1α and its target genes of Bcl-2 and SOD2 (Long et al., 2020). It has been reported that roxadustat enhanced its target genes of anti-oxidative enzymes SOD2 and HO-1 in cardiomyocytes, which could protect mitochondria *via* anti-oxidative action (Long et al., 2023). Roxadustat treatment remarkably ameliorated hypertension and organ injury, possibly through stabilizing HIF-1α and subsequently targeting eNOS, AGTR1, AGTR2, and oxidative stress (Yu et al., 2021). Given that roxadustat has shown these protective effects on myocardium by regulating energy metabolism、oxidative stress、apoptosis、and inflammation which are crucial for the development of DCM. This is a reasonable inference that we can explore deeper about roxadustat protective effects on DCM.One study showed that it takes time to upregulate the expression of HIF-1α and its downstream genes to achieve effective myocardial protection (Ohashi et al., 2020), which also reminds us that the myocardial protective effect of roxadustat may also vary depending on the mode of administration such as pretreatment or post-treatment.

### Roxadustat and diabetic nephropathy

Diabetic nephropathy (DN), affects up to 40% of patients with diabetes and is one of the leading causes of end-stage renal disease (ESRD), representing substantial social and economic burden worldwide (Gross et al., 2005; Pereira et al., 2022). DN often occurring at the end stage of DM, and effective methods to prevent and slow disease progression are still lacking. There are multiple factors participate in DN progression, such as apoptosis、oxidative stress、mitochondrial dysfunction and inflammation. Glomerular endothelial cell damage plays an important role in the occurrence and development of DN, and endothelial cell apoptosis is significantly increased in DN (Zhang X. et al., 2022). High glucose concentrations repressed HIF-1 in kidneys of animals with diabetes, through a HIF-PHD—dependent mechanism. The restoration of HIF-1 function attenuated ROS overproduction despite persistent hyperglycemia, and conferred protection against apoptosis and renal injury in diabetes (Zheng Y. et al., 2022). The prominent manifestation of DN kidney damage is the disturbance of oxygen metabolism. Considerable evidence has shown that hyperglycemia can cause oxidative stress damage in renal tissue by inducing excessive mitochondrial ROS production (Thangarajah et al., 2009; Miyata and de Strihou, 2010; Miyata et al., 2013). In recent years, many studies have shown that renal hypoxia is an indispensable pathological mechanism of DN, which usually leads to tubular and glomerular functional impairment (Mimura and Nangaku, 2010; Persson et al., 2012).HIF is an important transcription factor regulating intrarenal oxygenation (Norman and Fine, 2006). And the specific deletion of renal tubular HIF-1α in DM mice exacerbates mitochondrial dysfunction and ROS accumulation, which in turn leads to renal tubular damage and diabetic kidney injury consequently (Jiang et al., 2020). It has been shown that in STZ-induced DM rats, activation of HIF can protect against the appearance of enhanced DM-induced renal oxidative stress as well as prevent and ameliorate the progression of DN (Allison, 2014). In addition, it is believed that inflammatory processes are also able to accelerate the development and progression of DN (Tuttle, 2005). According to the previous studies, DN was accompanied by the increased amount of pro-inflammatory cytokines and chemokines, including tumor necrosis factor-α(TNF-α), interleukin (IL)-1βand IL-6 (Lim and Tesch, 2012).It has reported that HIF-PHIs can reduce inflammation and oxidative stress in chronic kidney disease (Mima, 2021). Roxadustat, a transcriptional activator of HIF, has been shown to ameliorate renal injury in cisplatin-induced DM mice by activating HIF-1α(Yang et al., 2018), and stable the expression of HIF-1α and HIF-2α to reduce high glucose-induced glomerular endothelial cell apoptosis (Xie et al., 2019).It has been shown that roxadustat slows the transition from ischemia-reperfusion-induced acute kidney injury to chronic kidney disease by finely coordinating HIF-1α/VEGFA/VEGFR1 signaling-dependent angiogenesis and the induction of HIF-1α/SOD2-dependent antioxidant defense capacity in a mouse model of ischemia-reperfusion acute kidney injury (Wu et al., 2021). Roxadustat pretreatment was anticipated to protect from FA-induced acute kidney injury (AKI) by stabilizing HIF-1α and therefore to strengthen the antioxidation system (Li et al., 2020c). Roxadustat protects against ischaemia/reperfusion-induced acute kidney injury through inhibiting the mitochondrial damage pathway in mice (Zhang M. et al., 2022). Roxadustat facilitates recovery from renal damage by ameliorating mitochondrial dysfunction induced by folic acid (Li X. et al., 2021). Roxadustat protects against renal ischemia/reperfusion injury by inhibiting inflammation (Miao et al., 2021). And it also reported that extracellular vesicles (EVs) derived from HK2 or HEK293 cells after roxadustat treatment could alleviate renal tubular injury and inflammation, suggesting a novel therapeutic role of roxadustat/EVs in the treatment of AKI (Ding et al., 2022). The pretreatment of roxadustat improved the creatinine elevation and renal tubular injuries induced by ischemia. What’s more, roxadustat protects against ischemia-induced AKI *via* improving CD73 and decreasing AIM2 inflammasome activation (Yang et al., 2022). These findings strongly suggested that roxadustat may be a new strategy for treating DN by regulating HIF-1α expression, inflammation, apoptosis, and oxidative stress. We urgently need to explore its influence on the above pathological processes in DN conditions.

### Roxadustat and diabetic retinal damage

Retinal tissue is one of the most metabolically active tissues in the body (Campochiaro, 2015). In diabetes, chronic hyperglycemia can result in retinal tissue metabolism disorders, vascular occlusion, and ischemic hypoxia, which can lead to diabetic retinopathy (DR) (Cheung et al., 2014; Zhang et al., 2018). DR is one of the most common microvascular complications of DM, it is a neurovascular disease (Cheung et al., 2010), retinal detachment (RD) can occur in the severe stage of DR, which is one of the major causes of visual impairment. Although the pathogenesis of DR has not been fully investigated, the main pathological changes associated with DR are the destruction of retinal capillaries and the formation of pathological blood vessels. The expression of HIF-1α was reported to be increased in the early stages of diabetic retinopathy in diabetic rats, and its expression level increased with the progression of the lesion in diabetic rats (Liu et al., 2015). In a diabetic animal study, the expression levels of HIF-1α, VEGFR2, p-mTOR and their downstream pathways were found to be significantly higher in the retinal tissues of rats in the STZ group than in the control group. In addition, the activity of HIF-1α and VEGF signaling pathways were significantly attenuated when mTOR was blocked by rapamycin. It is suggested that mTOR may be involved in the development of DR by upregulating the expression of HIF-1α(Wei et al., 2016). Studies have shown that VEGF plays a key role in the angiogenesis and proliferation of DR (Qaum et al., 2001). VEGF is located downstream of HIF signaling pathways and can be activated by HIF under hypoxic conditions (Fu et al., 2018), causing a significant upregulation of its expression level. VEGF, as a major factor in angiogenesis (Ferrara et al., 2003), promotes angiogenesis by improving blood flow and endothelial cell signaling (Crivellato, 2011). The disruption of the blood-retinal barrier (BRB) is a major pathogenic factor in DR, and increased VEGF in DR makes BRB disruption worse (Marfella et al., 2004). In a rat model of RD, roxadustat through activating the expression of HIF-1α which in turn upregulates the expression level of the target gene BNIP3, enhancing mitophagy and reducing ROS production, protecting photoreceptors from apoptosis and tissue damage after RD (Liu et al., 2016). The roxadustat-mediated HIF/BNIP3 pathway exhibited significant protective effects against RD which occurs in the end stage of DR. Roxadustat can prevent retinopathy of prematurity (OIR) by directing retinal HIF stabilization or indirecting hepatic HIF-1 stabilization (Hoppe et al., 2016). Importantly, roxadustat demonstrated a weak induction effect on Müller cell HIF-2α, which is the main mediator of retina pathologic angiogenesis, thus guaranting the safety when using this drug in ROP(Hoppe et al., 2020). The above findings provide more room for expanding PHI inhibitors for RD and also suggest that the progression and severity of DR should be taken into consideration when roxadustat is used to treat diabetic retinal damage.

### Roxadustat and diabetic wound healing

Impaired wound healing, especially in diabetic patients, can lead to considerable morbidity and mortality and may have a significant socioeconomic impact (Callaghan et al., 2008; Li G. et al., 2021). The diabetic wound healing process is slower than in non-diabetics. Foot ulcers are very common among diabetic patients and are one of the most serious complications of diabetes, with approximately 15% of diabetic patients suffering from diabetic foot ulcers (DFU) and more than 15% of DFU patients having to undergo surgery for subsequent amputation, which can lead to a relatively high mortality rate (Da Silva et al., 2021). The survival rates of DFU patients at 1, 2 and 5 years are 80.80%, 69.01% and 28.94%, respectively, which seriously affect the quality of life and even threaten life, and bring heavy burden to families and society (Brennan et al., 2017). Many studies have shown that elevated blood glucose levels in diabetic patients are a major cause of impaired angiogenesis and delayed wound healing (Piconi et al., 2006). And it has been shown that hyperglycemia can disrupt the stability of HIF-1α through a von Hippel-Lindau tumor suppressor protein-dependent mechanism, resulting in HIF-1α destabilization and inhibition of its downstream genes, especially VEGF expression, which is a central pathogenic mechanism for the delayed healing of diabetic wounds (Botusan et al., 2008). One study confirmed that early angiogenesis/neovascularization was promoted by enhanced HIF-1α/vascular endothelial growth factor expression in a male ICR diabetic mouse model, effectively promoting diabetic wound healing (Li Y. et al., 2020). It has been found that both fibroblasts isolated from T2DM patients and normal fibroblasts chronically exposed to high glucose showed inhibition of HIF-1α expression, which in turn led to a decrease in VEGF expression, resulting in reduced angiogenesis (Thangarajah et al., 2009; Thangarajah et al., 2010). In a DFU mouse model, it was found that promoting HIF-1α signaling may benefit both fibroblasts and endothelial cells, thereby promoting angiogenesis and wound healing through two pathways (Huang et al., 2020). The feasibility of treating diabetic wounds by blocking the interaction of HIF-1α with VHL in a diabetic mouse model has been verified (Li, Ko*,* et al., 2021). Researchers have demonstrated that roxadustat promotes angiogenesis through activation of the HIF-1α/VEGF/VEGFR2 pathway in STZ-induced DM rats, which indicates that roxadustat has a certain therapeutic effect on the healing of DM wounds (Zhu et al., 2019). It also reported that intraperitoneal injection of roxadustat effectively increased HIF-1α expression in skin of adult male BALB/c mice and accelerated the wound closure (Tang et al., 2018). In existing studies, roxadustat may have a therapeutic effect on wound repair by protecting HIF-1α from degradation. Meanwhile, considering the interaction between DM complications, the promotion of roxadustat on VEGF expression may affect the development of other complications, so the efficacy and effects of roxadustat may be different in different organ tissues. And selectivity of roxadustat for treating different complications should be considered in clinical application.

## Discussion

DM is the third most common non-infectious disease after cardiovascular disease and oncology. Globally, the number of people with diabetes has quadrupled in the past 3 decades, making it.

The ninth leading cause of death, with one in 11 adults worldwide suffering from DM(Zheng et al., 2018). DM, especially related complications, brings a heavy burden to the society and economy and seriously threatens human health. Hypoxia plays a crucial role in the development of diabetic-related complications. Under hypoxia condition, the body promotes the expression of downstream genes (VEGF, GLUT1, LDHA, e.g.) by activating the transcription of HIF-1α, thereby reducing the production of mitochondrial ROS and promoting tissue glycolysis to protect the body from oxidative stress, and maintaining the stability of metabolic levels. However, because the pathogenesis of DM is complex and the degree of hypoxia varies in different tissues, research on the treatment of diabetes and its complications by improving tissue hypoxia is still under further investigation. Roxadustat has been approved for the treatment of CKD anemia in Europe, China, and Japan, while other HIF-PH inhibitors have been approved in China and Japan alone (Macdougall, 2022). Comparing with other PHD inhibitors, roxadustat is generally well-tolerated and has been used in clinical practice. Furthermore, the convenience of oral administration for roxadustat, supported by the high treatment compliance observed in clinical trials, may provide an advantage for patients with CKD who are on peritoneal dialysis or not on dialysis, reducing the need of frequent hospital visits (Akizawa et al., 2020b). Roxadustat can also increase Hb to a level similar to that achieved by an ESA without substantially increasing the risk of cardiovascular events (Liu et al., 2020). The active role of roxadustat in regulating mitochondrial function, metabolic stability as well as apoptosis and inflammation, pathological processes that are also central to diabetic complications, has greatly encouraged us to further explore its protective role in diabetic complications. Many potential side effects of roxadustat should be seriously considered in the treatment of diabetic-related complications. A document released by the U.S. FDA on its official website on 15 July 2021 questioned the cardiovascular safety of roxadustat. In particular, for dialysis dependent (DD) patients, roxadustat had a higher cardiovascular risk than epoetin alfa; for NDD patients, roxadustat had a higher cardiovascular risk than placebo. Therefore, the FDA refused to approve the listing of roxadustat in the United States (Zhu et al., 2022). In two phase 3 studies in Japanese patients with anaemia and dialysis-dependent CKD, the most common treatment-emergent adverse events (AEs) with roxadustat included nasopharyngitis, back pain, diarrhoea, vomiting (Dhillon, 2019; Akizawa et al., 2020a). In addition, HIF functions in the progression of polycystic kidney disease (PKD), and roxadustat may promote the growth of renal cysts (Liu et al., 2021). Roxadustat inhibits the degradation of HIF-1α and thus increases its expression level, but targeting HIF-1α too late in the atherosclerotic process could trigger the rupture of fragile plaques, leading to disastrous consequences (Sluimer and Daemen, 2009; Jain et al., 2018). The enhanced pro-angiogenic effect of roxadustat during the treatment of diabetic cardiovascular disease and wound repair has the potential to exacerbate retinal damage. The main side effects of roxadustat were hyperkalemia、nasopharyngitis and fatigue compared with metformin, a common diabetes drug (Liu et al., 2020).In addition, the activation of HIF signaling may also be theoretically oncogenic, with excessive neovascularization in cancerous tissues that can promote tumor growth, metastasis and also present a barrier to tumor treatment. Long-term clinical data concerning potential adverse effects of HIF activation on CKD progression and cystogenesis are pending (Haase, 2009; Kraus et al., 2018).

## Conclusion

Overall, roxadustat has a potential on the alleviation of diabetes-related complications, but the exact molecular mechanism and dose-dependent relationship should be further clarified. The effect of roxadustat is HIF subtype-dependent, and the activation of different subtypes of HIF on the same disease may be different. It is also worth noting that roxadustat does not only have a positive potential on DM complications but also has certain side effects, which are related to the type of complications, the duration, severity of hypoxia and the activated pathways, etc. A reasonable improvement of these side effects would be beneficial to expand the clinical application of roxadustat.
